# Editorial: Prevention and control of human T lymphotropic viruses 1 and 2 (HTLV-1/2)

**DOI:** 10.3389/fmed.2022.998431

**Published:** 2022-08-11

**Authors:** Luiz Fernando Almeida Machado, Antonio C. R. Vallinoto, Carolina Rosadas, Graham Philip Taylor, Ricardo Ishak

**Affiliations:** ^1^Laboratório de Virologia Do Instituto de Ciências Biológicas da Universidade Federal Do Pará, Belém, Brazil; ^2^Section of Virology, Department of Infectious Disease, Imperial College London, London, United Kingdom

**Keywords:** HTLV-1, HTLV-2, prevention, control, prevalence, surveillance, public health

## Introduction

This research topic is kindly dedicated to the memory of our beloved and inspiring friend Professor Carlos Mauricio Castro Costa. It was interesting to see that, during the emergence of a new virus which has plagued humanity for 3 years now, Human T lymphotropic virus 1 (HTLV-1) received a lot of attention from health authorities, including the World Health Organization (WHO). This was a direct response to an instigative letter calling for action to eliminate the virus (1, 2). HTLV, which is the first human retrovirus described, approximately 40 years ago, has been extensively investigated with regard to its biology, diagnosis, prevention and to a lesser extent clinical impact. There are serious reasons to advocate in favor of the elimination of HTLV, considering the burden of most diseases presently associated with the infection (3). The response to the initiative of putting together a Research Topic dedicated to the “Prevention and Control of HTLV-1” showed a high demand, receiving attention from investigators from several countries in five continents. The issue received a diverse cast of manuscripts including classical and molecular epidemiology among several vulnerable population groups, diagnostic methods focused on prevention and control of the infection, the associated diseases and relevant co-infections. Furthermore, the importance of multidisciplinary care for those infected and affected was the subject of several manuscripts whilst the presentation of highly relevant public health policies already implemented and many others that are still in need to be further exploited completed this collection.

## HTLV prevalence, including vulnerable communities

The Research Topic received interesting papers in regard to the prevalence of HTLV-1 and subtypes found among vulnerable communities. Bandeira et al. designed an investigation that showed a low (0.4%) seroprevalence of HTLV-1 infection among prisoners from 12 closed prisons in Mato Grosso do Sul state, Central Brazil, with the predominance of the Cosmopolitan subtype Transcontinental subgroup. In contrast, Abreu et al. identified a high prevalence (3.0%) of HTLV infection among the Warao Indigenous Refugees in the Brazilian Amazon, with the presence of HTLV-1 subtype 1A (Cosmopolitan) and the Transcontinental subgroup and HTLV-2b, highlighting the need to create public health policies for the population of immigrants running from their own country to the Brazilian territory. Important information came from quilombos (slave remnant communities) in the state of Pará, Brazil where Brito et al. described a prevalence of 0.5% of HTLV (HTLV-1 and HTLV-2), reinforcing the need for infection control policies among these communities. Oliveira-Filho et al. investigated intra-family transmission among injecting drug users in a large geographical area of the state of Para and both HTLV-1 and HTLV-2 were found, again demonstrating the urgent intervention for infection control and prevention to reduce the spread of HTLV. A complex epidemiological model was set by Miranda C et al. to investigate a 10-year (2007–2016) analysis of HTLV-1/2 infection in first-time blood donors from four blood banks in Brazil. This revealed that HTLV-1/2 prevalence was not decreasing with a trend observed toward an increase in HTLV-1/2 infection among younger people, males, white skin color and higher education.

## Co-infections

Pereira et al. reported that in one of the most important epidemiological settings in Brazil, the state of Bahia, the prevalence of HTLV infection in people living with HIV-1 (2.4%) was much higher than among people with negative HIV-1 serology (0.5%), and that the highest frequency was among women. Three interesting and comprehensive reviews were presented. A systematic review by Ye et al. showing the importance and higher frequency of *Strongyloides stercoralis* infection among people living with HTLV-1 than in HTLV-1 seronegative and a strong association between severe strongyloidiasis and HTLV-1 infection. This was also reported by Montaño-Castellón et al. who also reported that HIV-1 and HTLV-1 coinfection and HIV-1 and HTLV-1/2 triple coinfection were associated with shorter survival, higher mortality rate, and faster progression to death, while coinfection by HIV-1/HTLV-2 seems to have neutral association with longer survival, slower AIDS progression, and lower mortality rate. The review by Rosadas and Taylor highlighted several factors related to the impact of HTLV-1 on co-infections and vice versa. Among other aspects, they emphasize that large scale prospective controlled studies on the prevalence and impact of HTLV-1 in co-infections are needed.

## HTLV diagnosis

Although there are multiple commercial assays suitable for screening, a repeated concern has been the need for cheaper tests to type and confirm HTLV infections. Franco et al. reported a multi-epitope protein, expressed in a prokaryotic system, including epitopes from Gag, Tax and Env proteins, aiming to develop a serological screening test for both HTLV-1 and HTLV-2, that showed great potential to be used to detect mono or co-infected individuals. Rocha Júnior et al. developed a promising, rapid, and sensitive duoplex-RT-PCR aiming to confirm and discriminate both HTLV-1 and HTLV-2. The new approach was validated by a multicenter study across Brazil. The assay presented adequate efficiency for HTLV-1/2 differentiation showing high sensitivity and specificity. Similarly, Gonçalves et al. developed and validated an in-house multiplex quantitative real-time PCR assay targeting the pol and tax genes. The laboratory tests demonstrated high sensitivity and specificity, leading the authors to conclude that their method is efficient and reliable for diagnosis. As a novel approach Machado et al. used the phage display to select HTLV-1 epitopes for diagnosis and described four clones with HTLV-1 mimetic peptides which aligned with gp46, protease and Tax, showing the potential of the technique for bioprospecting HTLV-1-related peptides with good potential either for diagnostic tests or as possible vaccine components for future studies.

## HTLV care

The care and management of persons with HTLV-1 infection is often complex and always multidisciplinary, starting with diagnosis. With the application of any diagnostic test comes the responsibility of the result, especially when a disease or the potential to develop disease is the diagnosed. Too often patients with HTLV-1/2 seroreactive results are sent away with anxiety and uncertainty. Lopes et al. describe the first-year results of implementing an HTLV-1 serodiagnostic service. Notably infection was confirmed and typed, all patients found to have HTLV-1/2 infection were referred to a counseling service and into follow up whilst two patients with suspected myelopathy were directed into neurological care, a model of care that should be provided wherever screening is introduced. Kimura et al. report the impact of HAM/TSP on health-related quality of life (HRQoL) in Japan with the interplay between the various elements of HAM contributing to impaired role functioning. They argue the case for comprehensive care to address the poor QoL. Meanwhile Aben-Athar et al. noted the absence of HTLV related care plans and therefore set out to develop and test a nursing care plan. The resulting detailed plan for care in the community aims not only to improve quality of life by tackling each of the many facets of HTLV-1-associated myelopathy but also to prevent avoidable deterioration such as falls leading to fractures which further impact pain, mobility and thus social participation and day to day living activities. From the same group, Sampaio et al. look at the problem from the occupational therapist's perspective identifying the key areas both in the home and the work environment that the team can help to address to reduce social exclusion, increase independence and thus improve quality of life. Galvao-Castro et al. continue this theme reporting on the importance of integrated care for people living with HTLV-1 and the difficulties experienced despite 20 years of an HTLV service. The importance of dedicated multi-disciplinary services is also picked up by Federico et al. not just in regions were HTLVs are common but also in non-endemic areas where knowledge is less. The impression of HTLV being invisible in their region of Argentina will resonate with persons living with HTLV around the world. Turning full circle this brings us back to the advice that is given to patients with HTLV. Often the evidence base for recommendations is limited, such as for HTLV management during pregnancy where there are no guidelines. Barr et al. take a pragmatic approach to this suggesting a management algorithm which can be tested and refined but for now provides a basis for prevention of mother to child transmission multi-disciplinary team (MDT) discussions. Miura et al. published an insightful review on the complexities of the molecular mechanism behind HTLV-1 persistence, gene expression and its clinical implications.

## HTLV transmission, prevention, and control

The mechanism of HTLV mother-to-child transmission *via* milk was reviewed by Millen and Thoma-Kress who highlighted the need to develop innovative strategies that prevent vertical transmission but allow safe breastfeeding. In this regard, promising results of an *in vitro* study was published by Schneiderman et al. The group elegantly showed that cabotegravir, the long-acting integrase strand transfer inhibitor, potently inhibits HTLV-1 transmission and suggested that it could be used to prevent HTLV-1 transmission. Bradshaw and Taylor reviewed available information on the effects of antiretrovirals on HTLV-1. They focused their work in the context of pre-exposure prophylaxis for HIV and its potential impact on HTLV-1 transmission. The authors reinforced the importance of conducting more studies in this promising area, being in accordance with current recommendations from WHO.

The state of the art and perspectives for the development of both preventive and therapeutic vaccines for HTLV-1 infection was addressed in the work by Tu et al. Regarding HTLV treatment, Gutowska et al. analyzed the potential of pomalidomide (Pom) treatment to reduce HTLV-1 viral burden using a rhesus macaque model. The hypothesis was that by increasing cellular surface expression, Pom would increase the susceptibility of HTLV-1-infected cells to NK and CTL killing. Indeed, Pom treatment resulted in immune activation, increase in specific humoral response and in the frequency of detection of HTLV proviral DNA. However, authors highlighted that Pom may not be effective as a single-agent therapeutic to control HTLV-1 infection.

## Public health policies

As a consequence of the recent initiatives from the WHO to coordinate efforts to control and eliminate HTLV-1, there was a second webinar sponsored by HTLV Channel/PAHO/WHO/Brazilian Ministry of Health, to celebrate the 2021 HTLV World Day and discuss public health policies worldwide. The webinar was covered by a report from Rosadas et al. highlighting the most relevant points from each presentation. Another interesting paper by Fowler and Einsiedel investigated the perception of patients and health personnel toward HTLV-1 in Australia, the geographical area with the highest prevalence of HTLV-1 in the world, affecting mainly the vulnerable aborigine population group and observed striking differences between community and healthcare workers. In a similar way, Martel and Gotuzzo, discussed and emphasized the importance of the sexual transmission of HTLV-1 and the relevance to the prevention and control of the virus. Miranda A. E. et al. prepared a novel approach to evaluate the public health policies in Brazil, using a methodology for strategic planning commonly used in business and economics. The SWOT (Strengths, Weaknesses, Opportunities, Threats) analysis, allows policy makers to shed light into the advances and the setbacks that need further attention. During the preparation of the present Research Topic, it became clear to the guest editors that we were facing a problem in regard to the name of the virus. Vallinoto et al. prepared a formal technical opinion to the HTLV academic community stressing the importance of using the formal nomenclature established by the International Committee on the Taxonomy of Viruses, ICTV, and the negative implications to refer to the virus other than Human T lymphotropic virus 1, in regard to its prevention and control, considering the present search systems commonly used in academic settings.

## Concluding remarks

Prevention and control are key points to achieve the ultimate goal to eliminate the circulation of an infectious agent and its associated diseases. So far, few viruses were successfully eliminated. HTLV-1 is a candidate for elimination despite the nature of the persistent infection that results in some chronic diseases as strategies that could achieve this are known. The manuscripts published in the present issue are highly significant as they provide important information that can be easily applied to many geographical areas in the world. Prevalence studies are still needed to support a comprehensive international discussion that will determine adequate methodological approaches and define targets for the prevention and control of HTLV-1/2. The contributions fulfilled the majority of the suggested subjects of the Research Topic, but there is still a need to develop economic analysis focusing on strategies to prevent and control HTLV infection.

We hope the data presented here will contribute to improve the quality of life of those living with HTLV and are applied halt further infections by HTLV-1/2.

## Author contributions

All authors listed have made a substantial, direct, and intellectual contribution to the work and approved it for publication.

## Funding

GT is supported by the Imperial NIHR Biomedical Research Centre. RI (#312979/2018-5), AV (#302935/2021-5), and LM (#314209/2021-2) are Research Grantees of the Conselho Nacional de Desenvolvimento Científico e Tecnológico, CNPq.

## Conflict of interest

The authors declare that the research was conducted in the absence of any commercial or financial relationships that could be construed as a potential conflict of interest.

## Publisher's note

All claims expressed in this article are solely those of the authors and do not necessarily represent those of their affiliated organizations, or those of the publisher, the editors and the reviewers. Any product that may be evaluated in this article, or claim that may be made by its manufacturer, is not guaranteed or endorsed by the publisher.
